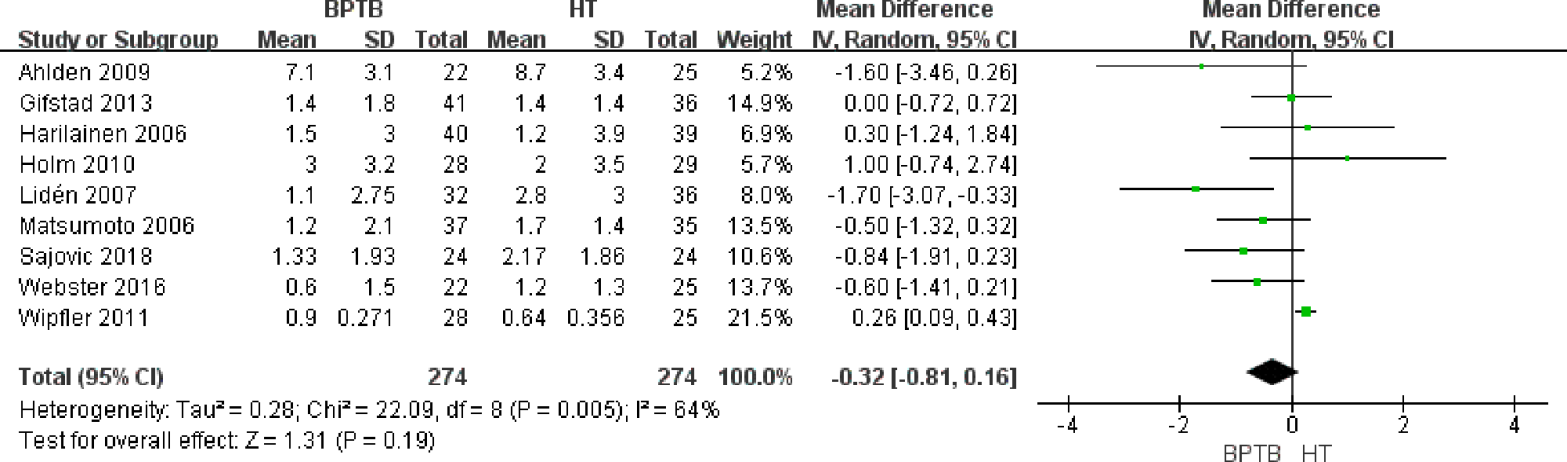# Outcome of bone-patellar tendon-bone vs hamstring tendon autograft for anterior cruciate ligament reconstruction: A meta-analysis of randomized controlled trials with a 5-year minimum follow-up: Erratum

**DOI:** 10.1097/MD.0000000000029873

**Published:** 2022-08-26

In the article, “Outcome of bone-patellar tendon-bone vs hamstring tendon autograft for anterior cruciate ligament reconstruction: A meta-analysis of randomized controlled trials with a 5-year minimum follow-up”^1^, which appears in Volume 99, Issue 48 of *Medicine*, figures 7 and 15 are the same figure. Figure 7 has been corrected to:

**Figure:**